# Response to Letter to the Editor Regarding the Article “Acute Myocardial Infarction and Stage E Shock: Insights From the RECOVER III Study”

**DOI:** 10.1016/j.jscai.2025.103599

**Published:** 2025-04-08

**Authors:** Ivan D. Hanson, Dana Bentley, Srihari S. Naidu, Mir B. Basir

**Affiliations:** a Department of Cardiovascular Medicine, Corewell Health William Beaumont University Hospital, Royal Oak, Michigan; b Abiomed, Danvers, Massachusetts; c Department of Cardiology, Westchester Medical Center and New York Medical College, Valhalla, New York; d Division of Cardiology, Henry Ford Hospital, Detroit, Michigan

The authors thank Drs Peled, Dau, and Jentzer for their thoughtful commentary^1^ on our manuscript, “Acute Myocardial Infarction and Stage E Shock: Insights From the RECOVER III Study,” published in *JSCAI*.^2^ They raise a potential concern that, in their view, patients in our analysis may have been inappropriately categorized as having Society for Cardiovascular Angiography & Interventions (SCAI) stage E shock and perhaps should have been categorized as stage C or D.

Peled et al contend that we categorized all patients with cardiac arrest as stage E. This is not true. As stated in the Methods section of the manuscript, we classified all patients with out-of-hospital cardiac arrest (OHCA) as stage E, which is consistent with other studies such as the Cardiogenic Shock Working Group^3^ and Microaxial Flow Pump or Standard Care in Infarct-Related Cardiogenic Shock (DanGer Shock).^4^ Although one could argue that a few OHCA patients may have a rapid clinical turnaround prior to reaching the hospital, thus being misclassified as stage E, this would be unlikely since only 10.4% of patients who present with OHCA in the United States survive to hospital discharge.^5^ On the other hand, we required patients with in-hospital cardiac arrest to meet ≥1 other stage E clinical criterion to be categorized as stage E. The other clinical criteria used to define stage E are pH ≤7.1, lactate ≥8 mmol/L, and/or profound hypotension despite multiple vasoactive medications.^6^ For reference, of the 82% of patients in our analysis that had cardiac arrest, 36.1% had OHCA and 57% had in-hospital cardiac arrest.

Peled et al further suggest that elevated blood lactate may falsely reflect shock severity, since a catastrophic event such as cardiac arrest may lead to a dramatic elevation in blood lactate level, but if the shock is promptly recognized and treated, lactate may rapidly normalize, and such a patient would be more appropriately categorized as stage C or D. Their criticism ironically emphasizes the importance of our study, which demonstrates the prognostic value of reassessing shock stage within 24 hours of the initial shock stage assessment. Decreasing blood lactate level, among other signs, defines improvement (or lack of improvement) in SCAI shock stage, which corresponds to improved outcomes.

SCAI shock staging has previously been validated in patients presenting with acute myocardial infarction and cardiogenic shock who were treated with a “best-practices” approach, which included Impella support and percutaneous coronary intervention.^7^ In that study, SCAI shock staging (C, D, or E) was found to have near-perfect interobserver reliability, and outcomes tracked closely with shock stage, so there should be no reason to question shock staging in the present study, as the methods used for staging were the same.

We can assure our readers that the stage E patients represented in this analysis were, in fact, stage E. As such, our work represents an important addition to the existing body of evidence, in which the “sickest of the sick” have been underrepresented. We acknowledge that, as with any classification tool, SCAI shock staging is not perfect. We certainly welcome future studies that would seek to further refine SCAI shock definitions, which in turn would enhance the care of patients across the spectrum of cardiogenic shock.
